# Understanding metabolic diversification in plants: branchpoints in the evolution of specialized metabolism

**DOI:** 10.1098/rstb.2023.0359

**Published:** 2024-11-18

**Authors:** Wenjuan Ji, Anne Osbourn, Zhenhua Liu

**Affiliations:** ^1^ Joint Center for Single Cell Biology; Shanghai Collaborative Innovation Center of Agri-Seeds, School of Agriculture and Biology, Shanghai Jiao Tong University, Shanghai 200240, People’s Republic of China; ^2^ Department of Biochemistry and Metabolism, John Innes Centre, Norwich NR4 7UH, UK

**Keywords:** gene duplication, enzyme promiscuity, biosynthetic pathway branchpoints, biosynthetic gene clusters, chemical diversity, specialized metabolism

## Abstract

Plants are chemical engineers par excellence. Collectively they make a vast array of structurally diverse specialized metabolites. The raw materials for building new pathways (genes encoding biosynthetic enzymes) are commonly recruited directly or indirectly from primary metabolism. Little is known about how new metabolic pathways and networks evolve in plants, or what key nodes contribute to branches that lead to the biosynthesis of diverse chemicals. Here we review the molecular mechanisms underlying the generation of biosynthetic branchpoints. We also consider examples in which new metabolites are formed through the joining of precursor molecules arising from different biosynthetic routes, a scenario that greatly increases both the diversity and complexity of specialized metabolism. Given the emerging importance of metabolic gene clustering in helping to identify new enzymes and pathways, we further cover the significance of biosynthetic gene clusters in relation to metabolic networks and dedicated biosynthetic pathways. In conclusion, an improved understanding of the branchpoints between metabolic pathways will be key in order to be able to predict and illustrate the complex structure of metabolic networks and to better understand the plasticity of plant metabolism.

This article is part of the theme issue ‘The evolution of plant metabolism’.

## 1. Introduction

Plants synthesize an enormous repertoire of specialized metabolites (sometimes also referred to as secondary metabolites or natural products). These molecules have important ecological roles in mediating interactions between plants and their environments. They are also of critical importance to humans for medicinal, agricultural and other industrial applications [1,2]. The ability of certain plant lineages to make particular types of specialized metabolites is likely a reflection of adaptation to different environmental niches [3,4]. Understanding the mechanisms of metabolic diversification that have led to the evolution of multi-step specialized metabolic pathways in the Plant Kingdom represents a major challenge.

From a biochemical perspective, the total of over 200 000 plant specialized metabolites reported so far can be roughly classified into a small number of major metabolite classes, mainly alkaloids, terpenoids, phenolics, sulfur-containing compounds and fatty acid derivatives [5,6]. The availability of a growing number of plant genome sequences is enabling rapid advances in our understanding of the types of molecules that plants produce, including the discovery of new metabolic genes and novel natural products [7]. These advances have raised fundamental questions concerning how new biosynthetic pathways arise and the molecular mechanisms underpinning the formation of different branches leading to novel metabolic routes. Gene duplication has frequently been proposed as a mechanism for the generation of the raw materials (i.e. spare copies of genes encoding enzymes that are free to functionally diverge) that enable diversification of biosynthetic pathways [8–10]. Enzyme promiscuity may also be a source of diversity generation, since natural selection may drive differentiation of enzyme properties in response to fluctuating ecological challenges [11,12]. As part of this review, we will consider the role of gene duplication and enzyme promiscuity in the evolution of emerging biosynthetic branches that lead to the expansion of metabolic diversity.

Investigations of metabolic diversification have typically focused on single enzymes rather than on the more complex and less tractable scenario of evolution of complete pathways. However there are numerous reports of complex plantspecialized metabolites with important medicinal or agronomic properties, the production of which appears to have originated through the joining of various distinct biosynthetic pathways [13–26]. The significance of these ‘joint efforts’ in the diversification of plant specialized metabolites has not been extensively reviewed. The hybridization of molecules that are derived from different biosynthetic routes is likely to have marked effects on chemical properties and biological activities, and strong natural selection is presumably required in order to maintain the newly formed biosynthetic route. Representative examples of this phenomena will be considered here.

In some cases, the genes for some or all of the enzymes of a plant specialized metabolite pathway are physically co-located in the genome like ‘beads on a string’ [3,27]. Such clustered pathways are commonly referred to as biosynthetic gene clusters (BGCs). One BGC potentially encodes a complete biosynthetic branch, thus providing perspectives on how chemical diversity in plants has expanded through specialized metabolic pathways. We aim to gain a deeper understanding of plant specialized metabolism by highlighting different branchpoints in the metabolic networks, their structural and evolutionary mechanisms, which underlie diversification, neofunctionalization and complexity of chemical diversity in plants. In addition, it may also inspire new approaches of mining biosynthetic pathways in a remarkable yet complex metabolic system.

## 2. Structure of plant metabolism: specialized metabolites and metabolic networks

Plant metabolism is usually divided into primary and secondary (or specialized) metabolism. Primary metabolism is for the most part conserved across diverse plant species, while the ability to produce distinct types of specialized metabolite is normally restricted to particular plant lineages [12,28]. Defining the exact borderline between primary and specialized metabolism is challenging since most plant metabolic pathways are as yet uncharacterized, and it is for the most part unknown how far conserved (primary) metabolism may go in terms of providing the precursors for specialized metabolism. Furthermore, analysis of the interface between primary and specialized metabolism is further confounded because the rates of flux through transient reactions are hard to measure and often require sensitive methodologies and finely controlled systems (e.g. isotope feeding) [29]. It is also the case that some specialized metabolites are known to have critical roles in fundamental processes such as development and reproduction in certain plant species [30,31], further obscuring the classical distinction between primary and specialized metabolism. Nevertheless, universal and essential building blocks such as sugars, amino acids, nucleotides, lipids, energy sources and hormones are typically considered as primary metabolites and are intrinsic to plant growth and reproduction. The metabolic pathways of primary metabolism and the molecules produced by these fundamental processes (e.g. glycolysis, the tricarboxylic acid (TCA) cycle, the pentose phosphate and shikimate pathways, amino acid biosynthesis) are in general very similar across plant species [5,32].

In contrast to primary metabolites, specialized metabolites are highly diverse, consistent with their various ecological functions in particular plant species. From an evolutionary standpoint, specialized metabolites are produced from different biosynthetic nodes of core primary metabolism (e.g. amino acids or acetyl-CoA), and their biosynthetic pathways have evolved in response to the ‘twists and turns’ of successive selection pressures [11,33]. Plant specialized metabolites are typically assigned to major compound classes according to their core chemical structures. These include phenolics (which share the presence of one or more phenol groups), alkaloids (mostly nitrogen-containing organic compounds), terpenes (which are derived from 5-carbon isoprene units), sulfur-containing compounds (e.g. glucosinolates in cruciferous vegetables), cyanogenic glycosides (featuring a nitrile moiety), highly modified sugars (e.g. acyl sugars in the Solanaceae), ribosomally synthesized and post-translationally modified peptides (RiPRs) and fatty acid derivatives [34–36] (table 1). It is important to note that this classification is based on general features of known specialized metabolites in plants, and does not encompass all branches of plant metabolism. Also, for a designated class of compound there is no general definition that covers all of the properties of the molecules encompassed within that group, a noteworthy example being the alkaloids [40].

**Table 1.** Representative classes of plant specialized metabolites.

class	core structure	biosynthetic routes	examples	references
phenolics	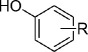	amino acid derivatives (phenylalanine, tyrosine)	flavonoids: flavone, phenolic acids, hydroxybenzoic acids; other phenolics: lignans	[34,35]
alkaloids	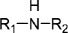	amino acid derivatives, nucleotide derivatives (adenine, guanine)	caffeine, betalains, morphine, strychnine, quinine, ephedrine nicotine	[32]
terpenes	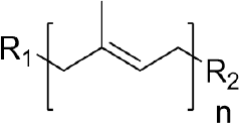	acetyl-CoA (to IPP DMAPP)	monoterpenes: carene; sesquiterpenes: longifolene; diterpenes: paclitaxel	[14,34,35]
glucosinolates	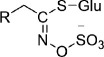	amino acid	glucobarbarin	[37]
cyanogenic glycosides	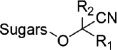	amino acid derivatives	prunasin, amygdalin	[38]
modified sugars	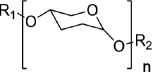	monosaccharides	acylsugars in Solanaceae	[35]
unusual peptides	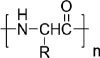	amino acid derivatives	RiPRs: pyrularia, viscotoxin B2, limenin	[39]
fatty acid derivatives	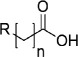	acetyl-CoA	falcarindiol, vernalic acid, ricinoleic acid	[35]

It has been proposed that plant specialized metabolism operates as a highly integrated network rather than as discrete linear pathways [41–43]. Impacts on one branch of the network may thus cause feedback regulation or flux redirection to another branch. As shown in *Arabidopsis thaliana*, the genetic knock-down of G and S monolignin biosynthesis redirects the metabolic flux to alternative branches of the network, resulting in ectopic accumulation of H-lignin, salicylic acid and flavonoids [44,45]. Similarly a null mutation gene encoding a cytochrome P450 that serves as the gateway enzyme for the biosynthesis of indole glucosinolates in *A. thaliana* (*CYP83B1*) perturbs the metabolic grid of indole glucosinolate and auxin biosynthesis, resulting in enhanced levels of free auxin [37]. Results from network analysis involving integration of multiple omics data provide further support to indicate that mutation of a component of one metabolic pathway often results in broad impacts on other pathways [46]. A recent study, again in *A. thaliana*, revealed that a triterpene metabolic network consisting of at least three pathways collectively produces more than 50 compounds. These compounds are synthesized in specific cell types in the root tips [47] and play important roles in shaping the root microbiome [48]. Given that many metabolic pathways operate as parts of integrated networks, it is not surprising that specialized metabolites are often present as mixtures of structurally related molecules in plant cells and in plant cell exudates. An improved understanding of the branchpoints between metabolic pathways will be key in order to be able to predict the consequences of metabolic perturbations and to better understand the plasticity of plant metabolism.

## 3. Generation of metabolic branchpoints by gene duplication and neo-functionalization

**Figure 1.**
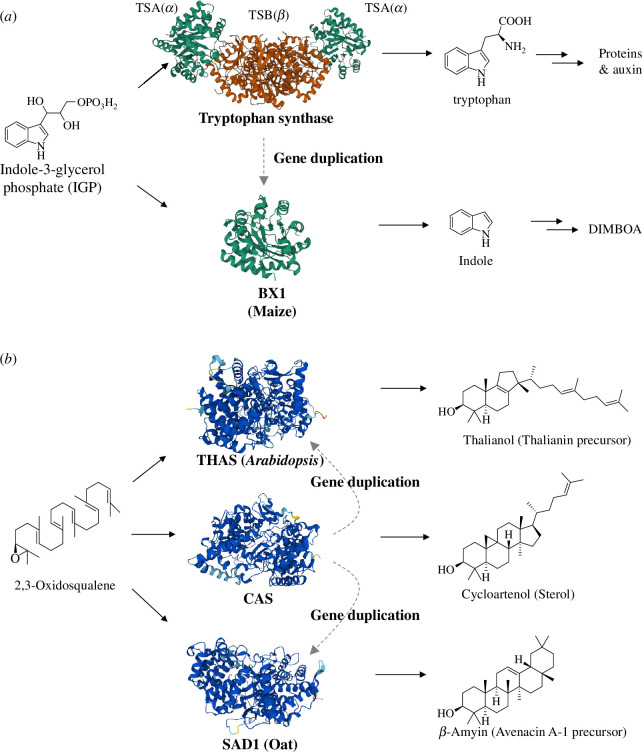
Generation of metabolic branch points by gene duplication. (*a*) The first step in the DIMBOA pathway (BX1) has been recruited from primary metabolism by duplication of the tryptophan synthase α-subunit gene TSA(α) [51]. (*b*) The genes encoding the oxidosqualene cyclases (OSCs) that catalyze the first committed steps in the biosynthesis of the specialized triterpenes thalianin and avenacin A-1, in *Arabidopsis thaliana* and *Avena strigosa* (diploid oat) respectively, have been recruited from the primary sterol pathway via gene duplication [27,55]. CAS, cycloartenol synthase; THAS, thalianol synthase; SAD1, β-amyrin synthase. Protein structures were predicted by AlphaFold2.

Gene duplication provides the raw materials for evolutionary innovation, enabling the birth of novel pathways [8–10]. After gene duplication the resulting gene duplicates may divide the ancestral functions (sub-functionalization) or one of the copies may evolve to gain a new function (neo-functionalization). If the descendent enzymes share the same substrate, a branch point will be generated. For example, tryptophan synthase (TS) in plants consists of two α- and two β-subunits that convert indole-3-glycerol phosphate (IGP) into l-tryptophan (TSA(α) and TSA (β), respectively) [49,50]. This is a two-step process in which TSA(α) converts IGP to indole, and TSA (β) then converts indole to l-tryptophan. BX1, the enzyme that catalyzes the first committed step in the pathway for biosynthesis of the plant defence compound 2,4-dihydroxy−7-methoxy-1,4-benzoxazin-3-one (DIMBOA) in maize, is thought to have originated by duplication of the gene encoding the tryptophan synthase α-subunit (*TSA*) [51,52]. l-tryptophan is a primary amino acid produced by all plants. However the duplication of *TSA* has created a unique branchpoint leading to production of DIMBOA, a specialized defence compound found only in maize and certain other plant species (e.g. wheat, rye) [51,53,54]. Both TSA and BX1 use IGP as their substrate, but they direct metabolic flux into primary and specialized metabolism, respectively (figure 1*a*).

2,3-Oxidosqualene is a universal precursor used by plants to make essential sterols, as well as a wide array of specialized triterpenes via a family of enzymes known as oxidosqualene cyclases (OSCs). The OSC that converts 2,3-oxidosqualene into the sterol scaffold cycloartenol (cycloartenol synthase, or CAS), is highly conserved across land plants [56]. However, examples of duplication and neo-functionalization of the *CAS* gene are widespread in plants, and OSCs collectively capable of generating over 200 different structurally diverse scaffolds have been reported [57]. The resulting scaffolds can be further modified by tailoring enzymes such as cytochrome P450s (P450s), acetyltransferases and glycosyltransferases, leading to >80 000 structurally and functionally diverse triterpenes [58]. Examples of these divergent OSCs include the *A. thaliana* OSC THAS [27], which produces the tricyclic triterpene thalianol, and the oat OSC SAD1 (also known as AsBAS1), which produces the pentacyclic triterpene β-amyrin [55]. Phylogenetic analysis indicates that *CAS* gene duplication events leading to the evolution of these two OSCs occurred separately in the eudicot and monocot lineages [27,52] (figure 1*b*). Duplication of diterpene synthase (*TPS*) genes has similarly been shown to produce branching points from the gibberellin biosynthetic pathway (i.e. primary metabolism), giving rise to alternative diterpene scaffolds that provide the starting point for a whole swathe of diversified (specialized) metabolism [52].

## 4. Generation of metabolic branchpoints through enzyme promiscuity

**Figure 2.**
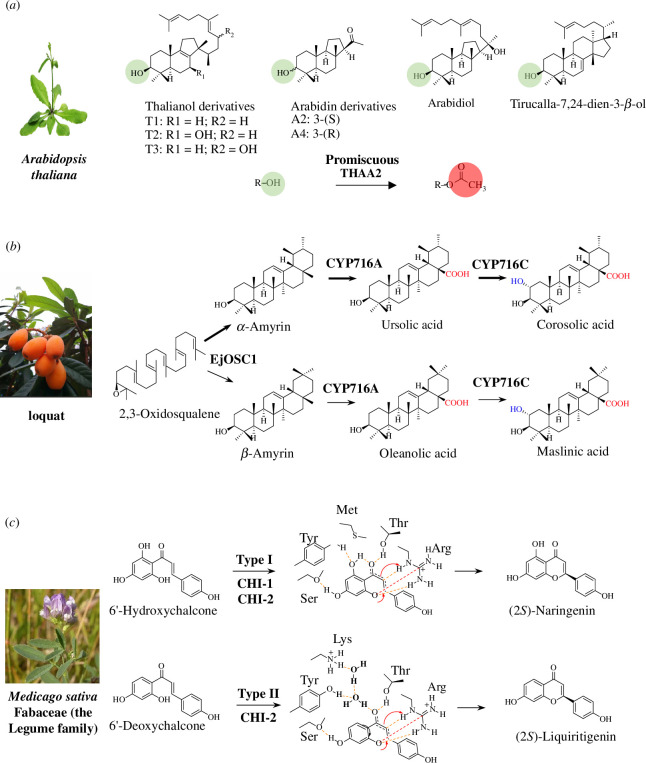
Metabolic branches that have arisen through enzyme promiscuity. (*a*) The acyltransferase THAA2 from *A. thaliana* is a promiscuous enzyme that is able to modify more than eight triterpene substrates, primarily thalianol and arabidin derivatives [48]. (*b*) The ursane- and oleanane-type triterpenes in loquat are produced by a suite of promiscuous enzymes. The triterpene scaffold enzyme EjOSC1 cyclizes 2,3-oxidosqualene into a mixture of α- and β-amyrin (95%:5%). The tailoring enzymes (EjCYP716A and EiCYP716C) are promiscuous, subsequently oxidizing α-amyrin and β-amyrin into corosolic acid and maslinic acid, respectively [63]. (*c*) Mechanistic elasticity of plant chalcone isomerases (CHIs). CHI-2s have evolved promiscuous substrate activation capability. A water-mediated hydrogen bond network is involved in CHI-2, differing from the activation of substrate by CHI-1.

A significant layer of complexity in plant specialized metabolism concerns enzyme promiscuity [59]. Specialized metabolic enzymes commonly recognize multiple substrates, a phenomenon that may interconnect multiple biosynthetic routes to form larger metabolic networks. This is often seen for tailoring enzymes such as P450s and BAHD acyltransferases. Members of the CYP716 family of P450s, for instance, are primary contributors to the diversification of pentacyclic triterpenes in plants [60]. These enzymes often recognize both α-amyrin and β-amyrin, isomers leading to ursane-type and oleanane-type triterpenes, respectively [61]. The BAHD family of acyltransferases is known to have a broad range of substrates, spanning almost every known class of compound in plants [62]. For example, the BAHD enzyme THAA2 catalyzes C3-acylation of more than eight triterpenes, so greatly diversifying pathways that shape the root microbiota in *A. thaliana* [48] (figure 2*a*).

On the other hand, enzyme promiscuity is reflected by observations that a single enzyme can produce multiple products [56,58]. For example the OSC BARS1 in *A. thaliana* produces the triterpene baruol as its predominant product but also catalyzes the formation of 22 other triterpene scaffolds [48,64]. Since most plant specialized metabolite pathways have not as yet been characterized, little is known about whether such side products may lead to branched pathways. A recent study on pentacyclic triterpenes in loquat (*Eriobotrya japonica*), however, suggests that a cascade of promiscuous enzymes is co-opted to take part in multiple pathways [63]. A multifunction OSC from this plant has been reported to synthesize a mixture of α- and β-amyrin (95%:5%). Interestingly, the CYP tailoring enzymes, which belong to the CYP716 subfamily (including CYP716A and CYP716C) were found to be multi-functional as well, and oxygenated both of these triterpene scaffolds [63,65] (figure 2*b*).

Understanding the catalytic permissiveness of enzymes for substrates or products is not trivial and is often hindered by the lack of structural enzymology and modelling evidence, capable of simulating the chemistry basis underscoring the enzyme promiscuity. A recent study has revealed that certain types of plant chalcone isomerases (CHIs) have evolved remarkable enzyme promiscuity by bifunctional substrate activation. Type-1 CHIs (CHI-1s) are ubiquitous across the green plant lineage and catalyze the cyclization of 6′-hydroxychalcones to form chiral flavanones such as (2S)-naringenin. Type-2 CHIs (CHI-2s), on the other hand, are more restricted and are most commonly found in the Fabaceae (the legume family). CHI-2s have comparable activity to CHI-1s for 6′-hydroxychalcones; however, CHI-2s also possess high activity for the more narrowly distributed 6′-deoxychalcones. Using comparative protein crystal structures and molecular dynamics simulations, this study demonstrated that CHI-1 and CHI-2 bind and promote productive substrate conformations through distinct chemical pathways. While both CHI-1s and CHI-2s were able to perform enantioselective oxa-Michael cyclizations on 6′-hydroxychalcones through Brønsted and Lewis acid interactions, CHI-2s utilized a water-mediated hydrogen bond network that enables expanded substrate reactivity for 6′-deoxychalcone [66] (figure 2*c*). The mechanistic elasticity of this example thus brings new insights into mechanisms of diversification of plant specialized metabolic pathways.

The evolutionary implications of enzyme promiscuity-generated pathway branchpoints are not well understood. It has been postulated that the permissiveness of an enzyme may encourage the retention of gene duplicates, which could favour subsequent functional specialization or neo-functionalization. This would, as a consequence, resolve the adaptive conflict caused by the promiscuous activities [67]. Such examples have been reported in the branching of lignin and flavonoid pathways [68,69]. From the perspective of population genetics, a minor metabolite may exhibit desirable activities in response to fluctuating abiotic and biotic ecological changes, thus resulting in retention and fixation of the multifunctional paralog [11]. Interplays between gene duplication and enzyme promiscuity are likely to be intricate, mirroring the remarkable complexity of specialized metabolic pathways.

## 5. Generating diversity through collaboration: merging different classes of specialized metabolites

There are many examples in which the conjugation of classes of compounds derived from independent biosynthetic routes has given rise to increased metabolic diversity and structural complexity. Where different classes of metabolites are combined to form a more complex compound, this can lead to the emergence of new branches from existing pathways, and the crosslinking of discrete pathways into a larger metabolic network. The ‘chimeric’ molecules generated in this way are likely to acquire new chemical properties (i.e. polarity, solubility and reactivity) and so constitute a new layer of chemical diversity. In addition, the multiple biosynthetic routes may span different subcellular organelles and so necessitate the need for transporters that enable the passage of intermediates between subcellular compartments and tissues.

**Figure 3.**
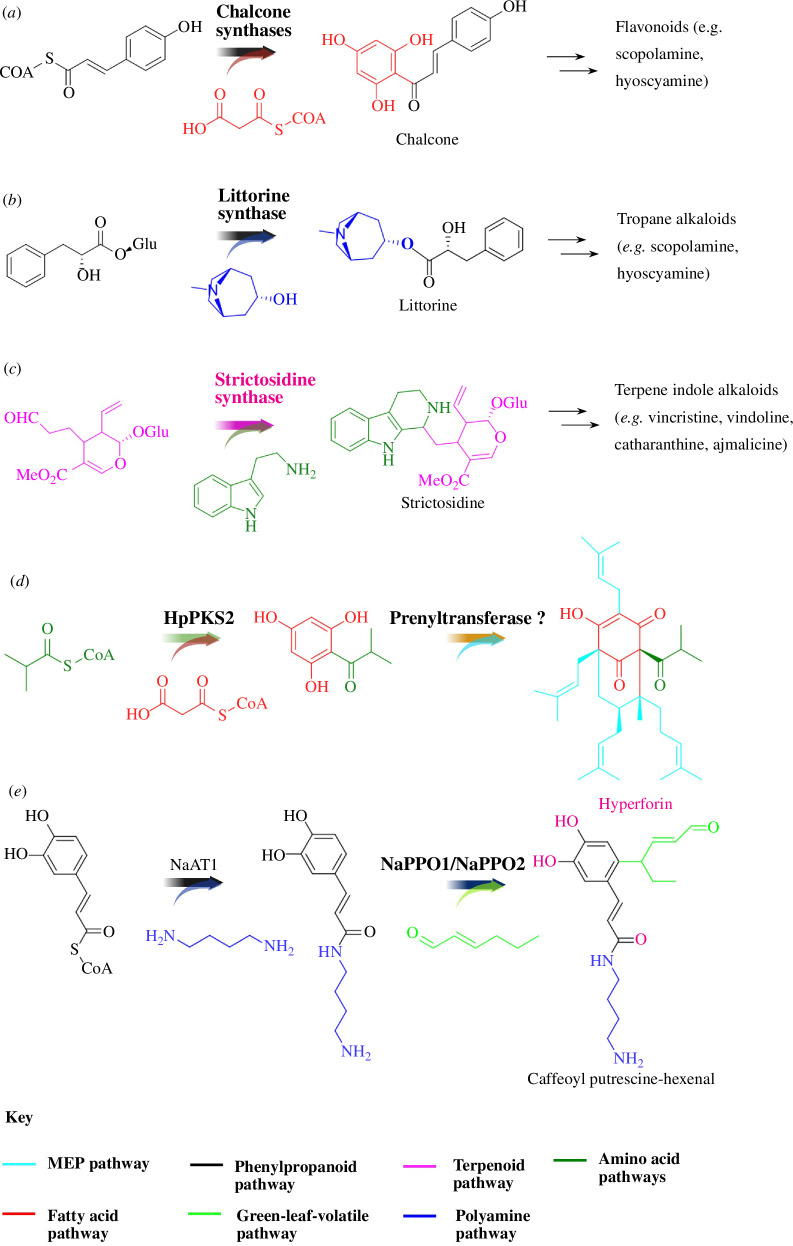
Metabolic diversity achieved by hybridizing multiple biosynthetic routes. (*a–c*) Representative examples for metabolite backbones that are made through the union of two distinct biosynthetic routes; (*d,e*) hyperforin (*d*) and caffeoylputrescine-hexenal (*e*) are the products of more than two classical pathways, representing greater chemical diversity. Pathways are modified from Wu *et al*. [70] and Bai *et al*. [71], respectively.

Phenylpropanoids are a vast and structurally diverse group of phenylalanine- or tyrosine-derived metabolites (table 1), the majority of which are fluxed into the biosynthesis of lignin, the second most abundant polymer on earth [23]. The diversity of phenolics in plants is mostly attributed to the expansion of phenylpropanoids, in part through convergence with other biosynthetic routes at various branch points. Flavonoids, are a consequence of an intersection between amino acid and fatty acid metabolism, using phenolic precursors and polyketide synthases (known as chalcone synthases, CHS) derived from fatty acid metabolism. CHSs catalyze a series of condensation reactions of three acetate units (C6C1 Claisen condensation). This fundamental branch provides the scaffold for thousands of flavonoids, including flavones, flavanols, isoflavones, flavanones and anthocyanins (figure 3*a*). Stilbene synthase, a similar enzyme to CHS, acts at the same branchpoint, utilizes the same substrates (*p*-coumaroyl-CoA and three molecules of malonyl-CoA), and catalyzes the same condensing type of enzyme reaction (resulting in sequential addition of acetate units via malonyl-CoA). However, it employs a newly formed ring system (alternative C2→C7 aldol condensation), which serves as the structural backbone for stilbene derivatives in some plants (72) [21,73] (e.g. resveratrol in grapes, and some other fruits and vegetables). Besides PKSs, acyltransferases are another exceptional family of ‘gluing enzymes’ that connect many biosynthetic routes into phenolic metabolism [18,23]. For example, phenolamides are specialized phenolics that combine with aliphatic or aromatic amines via BAHD acyltransferases and can be found in the pollen coat of eudicots [23,74]. BAHD acyl transferases are CoA-dependent enzymes that transfer various acyl-activated CoA thioesters to acceptor molecules. Another class of acyltransferases, namely serine carboxypeptidase-like (SCPL) acyltransferases, use acyl sugar donors and also serve to link different pathways. For example, littorine is a key intermediate in the biosynthesis of several important tropane alkaloids, including hyoscyamine and scopolamine. Combination of phenylpropanoids with tropine to form littorine is catalyzed by an SCPL acyltransferase [20] (figure 3*b*).

The production of terpene indole alkaloids (TIAs) is another outstanding example of the connection of multiple pathways. TIAs are a diverse class of natural products with a wealth of biological activities, including anticancer, -malarial and -arrhythmic activities. The backbone of TIAs is composed of tryptamine and secologanin, which are derived from the tryptophan and iridoid terpene pathway, respectively. The union of these two molecules to make the core structure is catalyzed by strictosidine synthase, which catalyzes a stereoselective Pictet–Spengler condensation between tryptamine and secologanin to yield strictosidine (iso-vincoside) [16,75,76] (figure 3*c*).

In some cases, the diversity and complexity of plant metabolism can be massively enhanced when more than two biosynthetic routes are integrated to produce natural products. One such example is hyperforin, a tetraprenylated polyketide from the medicinal plant *Hypericum perforatum* (St John’s wort), commonly used as an antidepressant drug [13,26,77]. The acyl chain of hyperforin originates from degradation of the amino acid valine followed by the formation of phloroglucinol PIBP via a type III polyketide synthase (PKS) activity [78], while the five isoprenoid units are derived from the plastid localized methyl-D-erythritol-4-phosphate (MEP) pathway [79,80] (figure 3*d*). Bai *et al*., using an omics-driven approach, recently identified a complex defensive compound, caffeoylputrescine-hexanal (CPH), which results from the metabolic union of three biosynthetic routes: the phenylpropanoid, polyamine and green-leaf-volatile pathways [71] (figure 3*e*). The integration of features from these different classes of specialized metabolites likely gives an additional ‘boost’ that contributes to the potent anti-insect properties of CPH. With advancements in the characterization of other complex specialized metabolic pathways, it is intriguing to consider the broader importance of this merging mechanism for generation of novel core structures for diverse natural products in plants.

## 6. Branchpoints associated with biosynthetic gene clustering

Over the last two decades, advances in genome sequencing and pathway elucidation have revealed over 40 examples of biosynthetic gene clusters (BGCs) in plants that encode pathways for a variety of different classes of specialized metabolites, including terpenes, cyanogenic glycosides, alkaloids, fatty acid derivatives and phenylpropanoids [81–83]. The features of these plant BGCs—genomic organization, potential mechanisms of assembly and evolutionary trajectories—have been extensively reviewed [83–86]. As for non-clustered metabolic pathways, the genes within plant BGCs appear to have been recruited directly or indirectly from primary metabolism.

**Figure 4.**
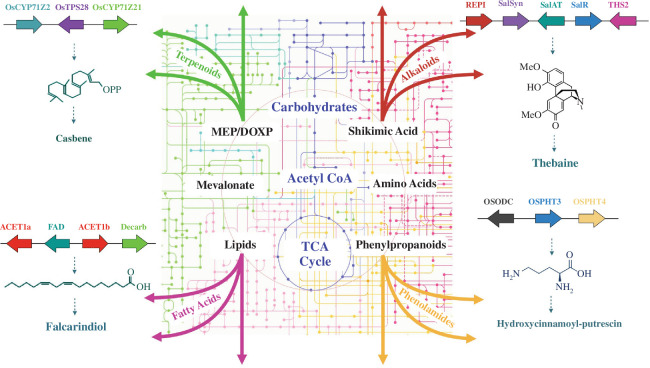
Biosynthetic gene clusters as branching points in plant metabolism. Biosynthetic gene clusters (BGCs), like other non-clustered specialized metabolic pathways, have arisen by recruitment of genes either directly or indirectly from primary metabolism. The distribution of particular types of BGCs is commonly restricted to closely related plant species, thus forming lineage-specific branches in plant metabolism.

The physical clustering of pathway genes into BGCs makes tracing and dating their evolutionary trajectories more convenient when compared to the genes for non-clustered pathways. Phylogenetic and syntenic analyses have revealed that particular types of plant BGCs are usually found only in a limited number of closely related species, often within a specific plant lineage [87], although a recent investigation of the miltiradiene diterpene gene cluster suggested that the core BGC genes are represented at the plant family level [88]. These observations strongly suggest that plant BGCs have evolved relatively recently in evolutionary time, thus defining new lineage-specific branches in plant specialized metabolism (figure 4). Comparative (pan)-genomics for the thalianol [89] and nepetalactone [90] biosynthetic pathways has further revealed that BGCs are dynamically evolving within and between plant species, rather than being a fixed feature of the genome. In Japanese catnip (*Schizonepeta tenuifolia*), the *p*-menthane BGC containing the first four steps of pulegone biosynthesis has an unusual bipartite structure, the core cluster (consisting of three genes) invertedly duplicated and separated by over 260 kb. Despite the mirrored feature of the two modules, the resulting six genes are highly co-expressed and functionally redundant [90]. This example thus suggests that BGCs or parts of BGCs can be recruited or incorporated as functional modules to form branches of plant metabolism. Interestingly, recent research has also provided evidence to suggest that in some cases plant BGCs can be transferred between distantly related plant species by lateral gene transfer, although the mechanisms underpinning this process are not known [70,91]. Taken together, the above examples exemplify the dynamic nature and potential significance of BGCs as ‘functional modules’ in driving the formation of new branches in plant metabolism (figure 4).

The phenomenon of physical clustering of biosynthetic pathway genes has led to the development of computation methods to mine plant genomes for the discovery of new candidate BGCs using algorithms such as plantiSMASH [92] and PhytoClust [93]. A complementary strategy involves focusing on a gene family for a particular type of scaffold-generating enzyme (e.g. OSCs), mining multiple closely related sequenced genomes, and using statistical methods to interrogate the genome neighbourhoods flanking predicted *OSC* genes to reveal enrichment of genes for different classes of tailoring enzymes [94]. While some plant BGCs contain the complete gene set for entire pathways (e.g. the 12-gene cluster for avenacin biosynthesis in oat [95], other pathways may be more ‘loose’ or ‘fragmented’ [87]. In some cases, the gene encoding the first committed pathway step may not be in the cluster. For example, QS-7, a saponin adjuvant identified from the soapbark tree, is synthesized via a 2,3-oxidosqualene cyclase (named OSC) scaffolding enzyme and multiple tailoring enzymes (CYPs, sugar transferases and an acetyl transferase). The core QS-7 biosynthetic genes, including the OSC and three CYPs, are not physically clustered with each other. However, a gene encoding one of the CYPs required for scaffold oxygenation (CYP716A297) is clustered with genes required for subsequent glycosylation steps [96]. In some cases, less strictly defined gene clusters generated via neo-functionalization of tandem gene duplications also contribute to the creation of metabolic diversity of plant specialized metabolism, particularly for genes encoding tailoring enzymes, presumably leading to new metabolic branches [97–99]. While not all plant specialized metabolic pathways exist as BGCs, clustering or partial clustering can greatly facilitate pathway discovery. When used in combination with gene co-expression analysis, which does not depend on physical clustering of genes in the genome, these two approaches collectively offer a powerful strategy for pathway elucidation [96].

## 7. Conclusions and perspectives

The enormous expansion of metabolic diversity is one of the key hallmarks of plant metabolism. By gaining a deeper understanding of the mechanisms that give rise to metabolic branchpoints in plants, it will be possible to advance our understanding of the structures, patterns and hierarchies that build up complex metabolic systems. From a chemical viewpoint, the vast diversity of plant specialized metabolism arises through two distinct presumably successive processes: firstly, the evolution of scaffold-generating transformations that utilize diverse but closely related substrates; and secondly, augmentation of these scaffolds with tailoring modifications (e.g. oxygenation, glycosylation, acylation). Precise details about the chemical and molecular logic underpinning metabolic diversification in plants have been extensively reviewed [100]. Mutations, gene and genome duplications, genetic drift and natural selection are frequently cited as major contributors at the genetic level, enabling branches for diversification of specialized metabolites that reflect adaptations to a specific environment [8–10]. At the enzymatic level, the promiscuity of enzymes for either substrates or products generates a suite of diverse natural products, thus laying the groundwork for novel metabolic pathways and contributing to metabolic diversity [11,12]. At the molecular structure level, distinct biosynthetic routes can be fused to yield greater levels of complexity, so generating a ‘chemical hub’ that can give rise to another tier of molecular diversity. In addition to the biosynthetic process *per se*, a variety of different regulatory systems may contribute to variation in metabolite content within and between individual cells, tissues and plant species, including modulation of gene expression, metabolite localization and transport. In order to attain a better understanding of plant specialized metabolism, it is thus necessary to consider not only biosynthetic genes and pathways, but also the mechanisms of temporal and spatial regulation that govern these, which could in turn give rise to novel phenotypic manifestations.

This review has summarized the key roles of metabolic branchpoints in the generation of molecular diversity in plants. Multi-omics driven approaches are routinely utilized to characterize novel enzyme functions and metabolic pathways [96,101,102]. The development of new genome mining approaches could open up opportunities to ‘fish’ for new nodes that may represent gateways to as yet unknown swathes of plant natural products. Advances in (pan)-genome sequencing are now enabling investigation of the relationships between genome structure, metabolic gene clustering and different ‘flavours’ of chemical diversity within and between different plant lineages [10,83]. There are about 40 clustered/partially clustered pathways now reported [83,85], roughly equivalent to the number of non-clustered plant natural product pathways that have been characterized. Branch-points associated with biosynthetic gene clusters have thus significantly facilitated plant metabolic pathway elucidation and characterization of novel enzyme functions. Although predictions of metabolic genes and pathways using artificial intelligence-based methods are still in their nascent stages, recent advances have successes in several important plants, including *A. thaliana* [103] and tomato [104]. In summary, the study of branchpoints in complex metabolic networks can provide important insights into our understanding of the expansion of chemical diversity in plants, and also opens up new opportunities to draw on the lessons learned to identify new enzymes and pathways involved in the diversification of specialized metabolites.

## Data Availability

This article has no additional data.
